# A Descriptive Study of the Demographic and Clinicopathological Characteristics of Individuals With Gallstone-Induced Pancreatitis in a Tertiary-Level Hospital in Ranchi, Jharkhand

**DOI:** 10.7759/cureus.57906

**Published:** 2024-04-09

**Authors:** Anil K Kamal, Praveenkumar A, Krishan Kumar, Kamlesh Kumar, Kumar Gaurav, Venkatesh N, Revathy P

**Affiliations:** 1 General Surgery, Rajendra Institute of Medical Sciences, Ranchi, IND; 2 Community Medicine, Rajendra Institute of Medical Sciences, Ranchi, IND; 3 General Medicine, Rajendra Institute of Medical Sciences, Ranchi, IND

**Keywords:** pancreatitis, gallstone, ranchi, outcome, clinicopathological characteristics, demographic, acute gallstone-induced pancreatitis

## Abstract

Background: Gallstones are a major cause of acute pancreatitis, which is associated with high recurrence, morbidity, and mortality. Careful consideration of demographic and clinicopathological features is required to understand the association between the cause and severity of pancreatitis in various populations, and such crucial information is lacking for Jharkhand’s population. Here, we sought to describe the demographic and clinicopathological features of gallstone-induced acute pancreatitis at a tertiary hospital in Ranchi.

Methods: This hospital-based descriptive study was conducted at Rajendra Institute of Medical Sciences in Ranchi. The hospital records of patients admitted to the surgical unit with acute gallstone-induced pancreatitis from January 2023 to December 2023 were analyzed. The study adhered to the Strengthening the Reporting of Observational Studies in Epidemiology (STROBE) guidelines.

Results: Of the 72 patients admitted with acute gallstone-induced pancreatitis (mean age: 42.5 years), 46 (64%) were males and 26 (36%) were females. All 72 patients had abdominal pain and 44 (61%) were vomiting. The severe vs. non-severe pancreatitis groups differed significantly in age (≥40) and male gender (p = 0.013 and 0.031, respectively). A total of 45 (62.5%) patients had severe gallstone-induced pancreatitis, and the most common complication was acute kidney injury, followed by pleural effusion (18 (25%) and 13 (18.1%) cases, respectively).

Conclusions: Our study revealed that gallstone-induced pancreatitis was more common in males and that age and gender were significantly associated with severity. However, late presentation to the hospital may have influenced our study, resulting in more severe cases being reported, with the most common complication being acute kidney injury. To our knowledge, this is the first study to describe the demographic, clinicopathological, and outcome data of acute gallstone-induced pancreatitis in Ranchi. These results can guide hospital policy development to improve patient outcomes.

## Introduction

Acute pancreatitis, an inflammatory process that occurs in the pancreas, is a common cause of acute abdominal pain [1]. Globally, acute pancreatitis incidence varies from five to 50 cases per 100,000/year [2]. Gallstones and alcohol abuse are the main drivers of acute pancreatitis, accounting for 70%-80% of the cases [3], although acute pancreatitis with idiopathic etiology that causes biliary microlithiasis or sludge accounts for a third of the cases [4].

To plan effective treatment modalities, reduce complications, and prevent recurrence, it is essential to determine the etiology of acute pancreatitis [5]. Because gallstones are a major cause of pancreatitis, it is crucial to understand the pathogenesis of gallstone-induced pancreatitis, which is unclear [5].

There are two accepted theories: the obstructive theory, in which pancreatic duct blockage causes increased pressure in the pancreatic duct because of pancreatic secretions [6], and the common channel or reflux theory, in which an impacted stone at the ampulla of Vater leads to bile drainage into the pancreatic duct, which may cause acinar cell necrosis [2].

However, in most cases, there is only transient obstruction, which is relieved after the passage of the stone into the duodenum [7]. If gallstone pancreatitis is missed at first, there is an increased risk of recurrence (60%) within six months [8], mortality (10%), and morbidity (30%-40%) [9].

Gallstone-induced pancreatitis is diagnosed based on clinical features, biochemical markers, or radiologic investigation. Although whole-abdomen ultrasound has a 95% sensitivity for gallstone detection, its gallstone and choledocholithiasis sensitivities are reduced to 67%-87% and 20%-50%, respectively, during acute pancreatitis [8]. A three-fold increase in the levels of serum alanine transaminase from the normal limit has been shown to have a 95% positive predictive value for gallstone-induced pancreatitis [10].

Although pancreatitis is usually managed conservatively, UK guidelines recommend that for severe gallstone-induced pancreatitis, endoscopic retrograde cholangiopancreatography (ERCP) with sphincterotomy should be considered within 72 hours [11]. The British Society of Gastroenterology and the American Gastroenterological Association recommend that for mild gallstone pancreatitis, cholecystectomy should be performed during the same hospital admission, and for all cases, delays should not exceed four weeks after hospital discharge [12,13]. Cholecystectomy was also reported to reduce recurrence by 1%-1.7% [13].

A careful consideration of demographic and clinicopathological features is required to understand the association between the cause and acuteness of pancreatitis in various populations [14] but this crucial information is lacking for Jharkhand’s population. Here, we sought to describe the demographic and clinicopathological features of gallstone-induced acute pancreatitis at a tertiary hospital in Ranchi.

## Materials and methods

This descriptive study was conducted at Rajendra Institute of Medical Sciences, Ranchi, and was approved by the Institutional Ethics Committee of Rajendra Institute of Medical Sciences (Memo No.: 139; dated: July 12, 2023). The study analyzed the hospital data of patients with acute gallstone-induced pancreatitis admitted to the surgical unit from January 2023 to December 2023.

Based on convenience sampling, all 72 patients admitted for acute gallstone-induced pancreatitis were included in the study. All participants were aged >18 years. Acute pancreatitis was diagnosed based on symptoms like acute abdominal pain with or without radiation to the back, a greater than three-fold increase in serum amylase and lipase levels when compared with normal levels, and radiological findings. The biliary cause of pancreatitis was confirmed based on an ultrasound suggestive of gallstones or a three-fold elevation in serum alanine aminotransferase (ALT) levels. A whole-abdomen computed tomography scan was done only when needed.

**Figure 1:**
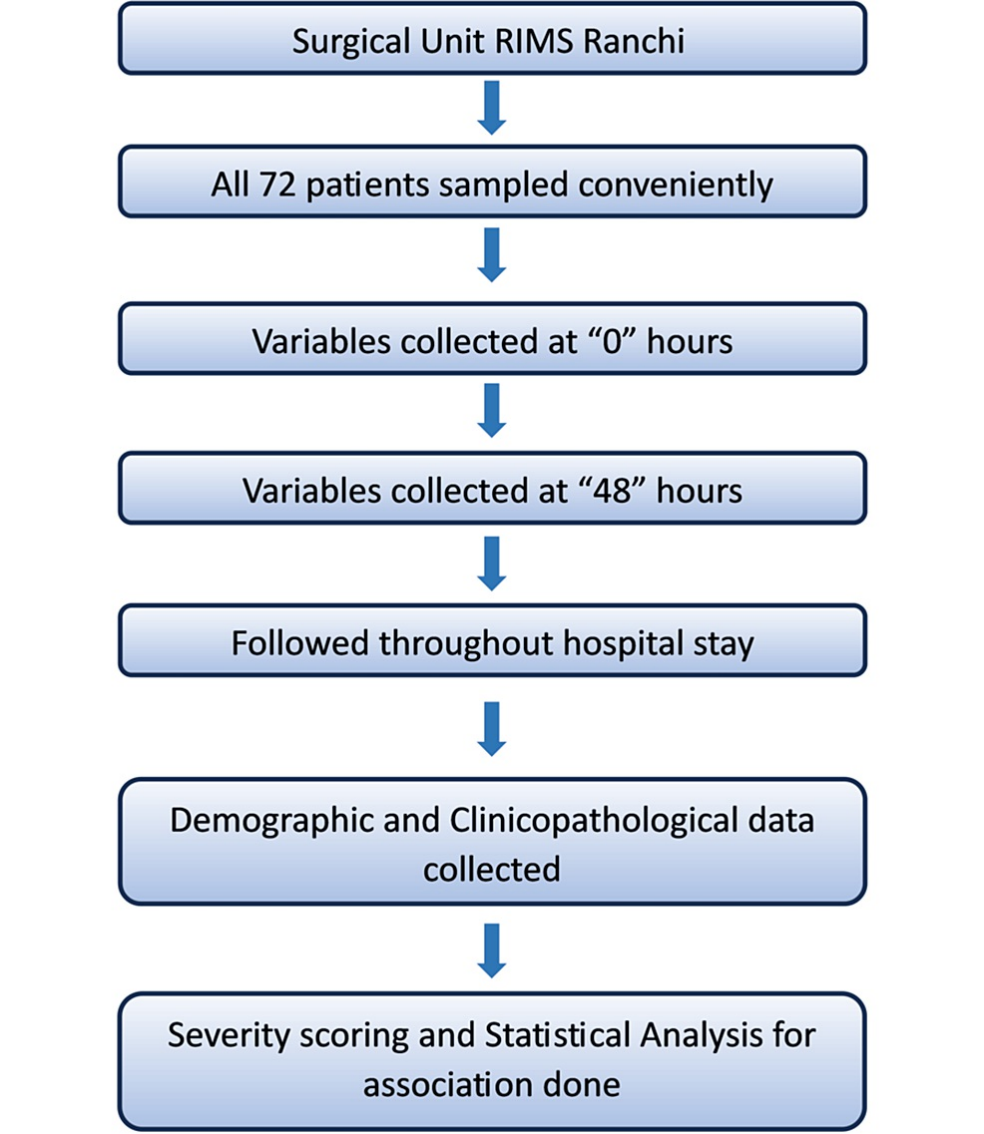
A flowchart of the study’s overall methodology. RIMS: Rajendra Institute of Medical Sciences.

Based on patient history or clinical examination, those with chronic pancreatitis, alcohol consumption, and pancreatitis because of other causes were excluded from the study. The study protocol was explained to all patients and proper written informed consent was taken. After obtaining a complete patient history and conducting a general and systemic examination, routine blood analyses (complete blood count, liver and renal function test, prothrombin time, arterial blood gas analysis, and serum electrolytes) were done at the time of admission and repeated after 48 hours. Vitals were monitored and recorded throughout the hospital stay. All information was gathered using a standard format. The variables of interest were age, gender, clinical presentation, supportive laboratory parameters, and disease-associated complications. All participants were followed up throughout their hospital stay. Because severity was a secondary outcome, a standard scoring system like APACHE II (Acute Physiology and Chronic Health Evaluation II) or Atlanta classification was not used, and disease severity was assessed based on Ranson scores, with scores ≥3 being considered severe. The participants were divided into the severe and the non-severe groups and then the association of severity with age and gender was evaluated. The study adhered to the Strengthening the Reporting of Observational Studies in Epidemiology (STROBE) guidelines. The study’s methodology is summarized in Figure 1.

Data analyses

Data were entered in a Microsoft Excel spreadsheet (student version 2021; Microsoft Corporation, Redmond, WA) and exported to SPSS version 27.0 (IBM Corp., Armonk, NY) for analyses. Continuous variables are presented as mean ± standard deviation. Categorical variables are presented as frequencies and percentages. Categorical variables were age group, gender, clinical presentation (abdominal pain, vomiting, fever, jaundice), elevated laboratory parameters, complications (acute kidney injury, pleural effusion, acute respiratory distress syndrome, necrotizing pancreatitis), and outcomes. Categorical variables were analyzed using a chi-square test.

## Results

**Figure 2:**
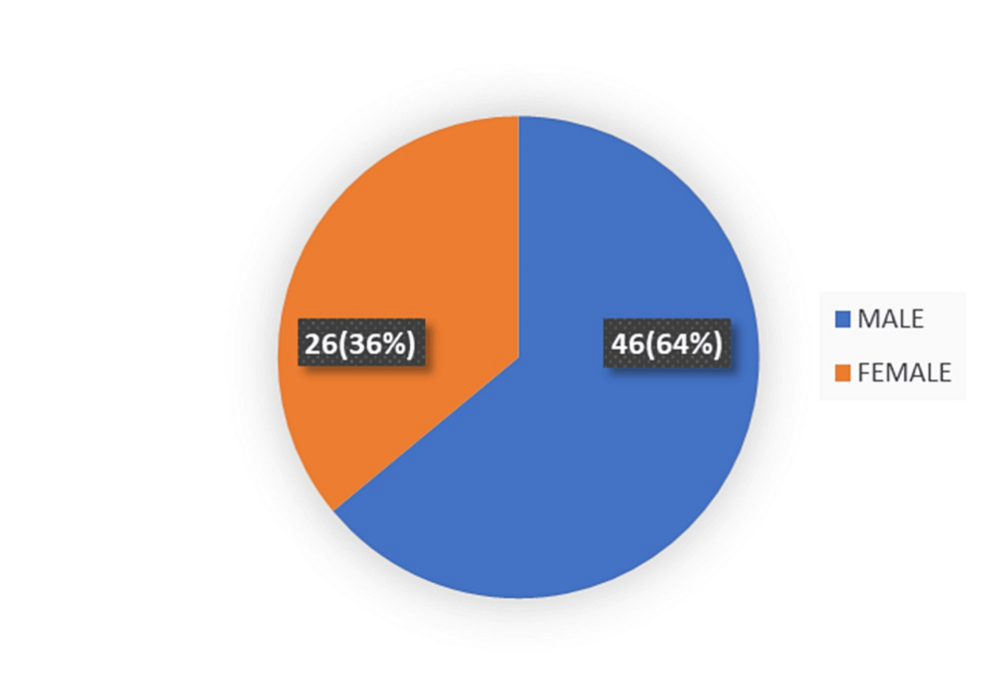
The number of male vs. female patients in the study group.

During the study period, 72 patients (male: 46 (64%); female: 26 (36%); male-to-female ratio: 1.7:1) were admitted with acute gallstone-induced pancreatitis (Figure 2).

The mean patient age was 42.5 years. Our analysis revealed that most patients were aged 20-40 years (Table 1).

**Table 1:** Disease prevalence across age groups (N = 72).

Age group (years)	Number of patients	Percentage (%)
<20 years	1	1.4
20-40 years	40	55.6
40-60 years	12	16.7
>60 years	19	26.4
Total	72	100.0

All patients had abdominal pain, 44 (61%) were vomiting, 22 (30.6%) had a fever, and 18 (25%) had jaundice (Table 2).

**Table 2:** Clinical presentation of patients with acute gallstone-induced pancreatitis.

Clinical presentation	No. of patients	Percentage (%)
Pain	72	100
Vomiting	44	61
Fever	22	30.6
Jaundice	18	25

Our analyses revealed that the levels of serum amylase and lipase were increased by threefold in 40 (55.5%) and 59 (81.9%) cases, respectively (Table 3).

**Table 3:** Elevated laboratory parameters.

Investigation	No. of patients	Percentage (%)
Serum amylase	40	55.5
Serum lipase	59	81.9

Table 4 shows demographic characteristics based on the severity of gallstone-induced pancreatitis. Age and male gender were significantly different in the severe and non-severe pancreatitis groups (p = 0.013 and 0.031, respectively).

**Table 4:** Demographic characteristics based on pancreatitis severity.

Characteristics		Severe acute pancreatitis		Non-severe acute pancreatitis		P-value
		N	%	N	%	
Age	<40 years	18	48.6%	19	51.40%	0.013
	≥40 years	27	77.1%	8	22.90%	
Sex	Male	33	71.7%	13	28.30%	0.031
	Female	12	46.2%	14	53.80%	

**Figure 3:**
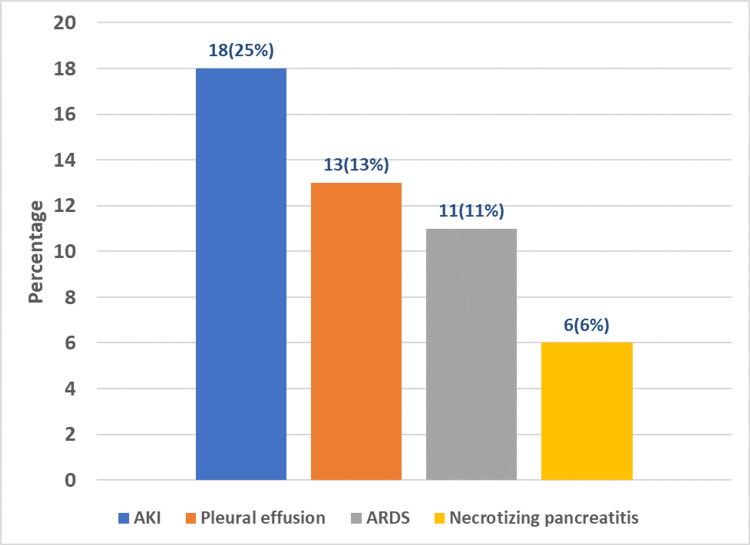
The complications of acute gallstone-induced pancreatitis. AKI: acute kidney injury; ARDS: acute respiratory distress syndrome.

Our analysis identified acute kidney injury (AKI, 18 cases (25%)) as the most common complication, followed by pleural effusion (13 cases (18.1%)) (Figure 3).

**Figure 4:**
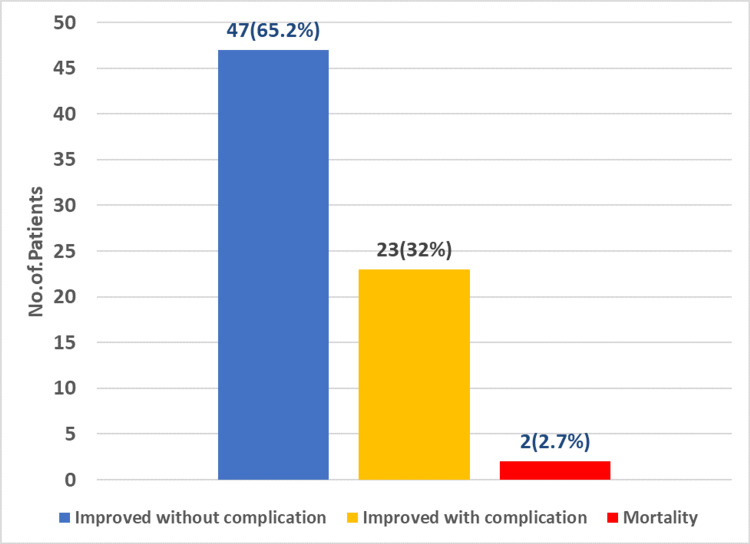
The outcome of acute gallstone-induced pancreatitis.

In this study, most of the patients (47) improved without any complications, whereas two patients died because of multi-organ failure (Figure 4).

## Discussion

In the last two decades, pancreatitis incidence has increased by about 30% [10] and gallstone-induced pancreatitis accounts for 50%-70% of the cases [3,14]. Although gallstones most frequently present as acute cholecystitis, many studies have shown that gallstone-induced pancreatitis carries a mortality rate of 2%-17% [10]. This descriptive study involved 72 patients diagnosed with acute gallstone-induced pancreatitis, and of these, 46 (64%) were males, whereas 26 (36%) were females (male-to-female ratio: 1.7:1). These findings contradict other studies that have shown a female predominance [9,10,13]. This may be because gallstone-induced pancreatitis is associated with transient obstruction only, and many times, it has a mild course [7] that subsides spontaneously without the need for tertiary-level medical care. The mean duration at which the patients presented to the hospital with the clinical features was four days, indicating that there is low awareness or other difficulties limiting people’s healthcare access in Ranchi. In this study, the participants’ ages ranged from 19 to 75 years (mean age: 42.5 years), which is consistent with the findings by Manandhar et al. [10], although the age is lower than the mean age reported for Western countries [9].

Our analysis revealed that most participants (N = 40, 55.6%) were in the 20-40 years age group, whereas the >60, 40-60, and <20 years age groups had 19 (26.4%), 12 (16.7%), and one (1.4%) participant, respectively.

At the time of presentation, all patients had abdominal pain, whereas 44 (61%) and 22 (30%) had vomiting and fever, respectively. These findings are consistent with those reported by Khanal et al. and Ramu et al. [14,15].

The diagnosis of gallstones-induced acute pancreatitis was based on the patients’ clinical presentations, as well as their levels of serum amylase, lipase, and ALT. Additionally, whole-abdomen ultrasound was used to aid in the diagnosis. In this study, serum amylase, lipase, and ALT levels were elevated threefold in 40 (55.5%), 59 (81.9%), and 53 (73.6%) cases, respectively, which is consistent with the findings by Manandhar et al. [10], and gallstones were detected in all patients.

Based on the Ranson criteria, severe acute gallstone-induced pancreatitis (indicated by scores ≥3) was detected in 45 (62.5%) of the participants, which is similar to the proportion (60%) reported by Manandhar et al. [10]. The high incidence of severe cases might be because of late presentation at our tertiary center. In this study, the most frequent complications in patients with acute gallstone-induced pancreatitis were acute kidney injury (18 cases, 25%) and pleural effusion (13 cases, 18.1%), which is consistent with the findings by Khanal et al. [14]. Although many studies recommend ERCP within 72 hours of an acute gallstone-induced pancreatitis attack and have shown that it decreases complications [11], this study’s participants were managed conservatively because ERCP facilities were unavailable. However, 23 (31.9%) participants with complications improved, whereas 47 (65%) had uneventful recoveries and only two (2.7%) died of complications that caused multi-systemic failure, which is consistent with the findings by Khanal et al. [14].

## Conclusions

This descriptive study revealed a male predominance in gallstone-induced pancreatitis and that age and gender were significantly associated with severity. The late patient presentation to our hospital may have influenced our findings, resulting in more severe cases being reported. However, this study has limitations, including a relatively small sample size and the limited number of CT scans performed. Additionally, interventions like ERCP with sphincterotomy, laparoscopic cholecystectomy, or open cholecystectomy were not performed during the same admissions. Nonetheless, to our knowledge, this is the first study to describe the demographic, clinicopathological, and outcome data of acute gallstone-induced pancreatitis in Jharkhand. Overall, given the similarities in institutional and population characteristics across developing countries, this study may indicate the condition in other developing countries. This study’s results will guide hospital policy development to improve patient outcomes.
